# 43-Year Temporal Trends in Immune Response to Oral Bacteria in a Swedish Population

**DOI:** 10.3390/pathogens9070544

**Published:** 2020-07-07

**Authors:** Anders Esberg, Anders Johansson, Rolf Claesson, Ingegerd Johansson

**Affiliations:** Department of Odontology, Umeå University, 901 87 Umeå, Sweden; anders.p.johansson@umu.se (A.J.); rolf.claesson@umu.se (R.C.); ingegerd.johansson@umu.se (I.J.)

**Keywords:** adaptive immune response, antibody, IgG, bacteria, oral, time trends

## Abstract

Bacteria colonizing the mouth induce an adaptive immune response with the systemic and local presence of species or strain-specific immunoglobulins. Few studies have addressed global antibody patterns for oral bacteria or potential population time trends. We assessed these aspects in relation to a panel of oral bacteria. Using multiplex immunoblotting, IgG levels for 26 oral bacterial species (54 strains) were determined in 888 plasma samples from 30-year-old early pregnant women (*n* = 516) and 50-year-old men and women (*n* = 372) collected between 1976 and 2018. Inter-species correlations were found and age-dependent profiles and levels of immune responses to oral bacteria confirmed. We found temporal trends in the global and single-species antibody responses, but this was age-specific with both inclining and declining shifts. Prominent shifts in the younger group increased IgG towards health-associated *Streptococcus salivarius* and *Streptococcus sanguinis*, and in the older group towards disease-associated *Aggregatibacter actinomycetemcomitans, Filifactor alocis,* and *Streptococcus mutans*, among others. We concluded that temporal shifts occurred from 1976 to 2018, which may reflect improved oral health (more remaining teeth) and altered lifestyle habits, but this needs to be evaluated in observational studies considering more aspects.

## 1. Introduction

Profiling of oral microbiota by cultivation and DNA sequencing has identified more than 700 bacterial species organized in spatially arranged, niche-distinct communities [1,2]. Most species in these communities are commensal bacteria, but some opportunistic species are associated with the development of the most common infection-induced oral diseases, periodontal disease and dental caries [3]. The most striking transformation from a healthy to a disease-associated microbiome is reduced diversity into a dysbiotic stage [4,5]. In the dysbiotic microbiota, some bacterial species and putative virulence factors are overrepresented in periodontitis, and others in caries [6,7]. In periodontal disease, *Aggregatibacter actinomycetemcomitans* and *Porphyromonas gingivalis* are key organisms with virulence factors linked to mechanisms involved in the pathogenesis of periodontitis, but a wider range of species have been indicated [8,9]. The obligate anaerobic Gram-positive bacterium *Filifactor alocis* has more recently been linked to periodontitis by acting in concert with other periodontitis-associated bacteria [10]. One major pathogen in caries is *Streptococcus mutans*, the virulence of which is associated with acid tolerance, avid lactate production, and adhesive properties [7]. However, several more species contribute to an acidic environment upon carbohydrate intake, and caries may even develop in the absence of *S. mutans*. Furthermore, the great genetic diversity within the different species in the mouth may include both harmless and virulent genotypes within the same species [7,11,12].

Bacteria colonizing various surfaces in the oral cavity induce an adaptive immune response, leading to a systemic and local presence of species, or even strain-specific immunoglobulins [13]. The immunoglobulins bind to bacterial protein epitopes on the bacterial surface, which results in decreased virulence and an impaired ability to invade the host tissues [13]. The protective role of these antibodies in oral infectious diseases is poorly understood, but their systemic presence may limit adverse effects during bacteremia. The levels of systemic antibodies against different oral bacterial species vary in regard to age, oral disease condition, and geographic origin [14,15]. In addition, lifestyle factors, such as smoking, food intake, and oral hygiene habits, may influence the net effect of oral bacteria on the adaptive immune response [16]. Based on current knowledge, the main oral microbiota colonization starts at birth and generally reaches maturation in adolescence [17]. The temporal trend in the abundance of disease-associated species, e.g., *A. actinomycetemcomitans* and *S. mutans*, is unknown, but there are indications that at least *S. mutans* has decreased in populations with organized dental care [18]. Such a trend shift can be speculated to reflect the impact of dental care per se or parallel lifestyle changes, such as the reduction in smoking seen in many countries [19,20]. However, the association between the immune response, i.e., immunoglobulin profile, and oral bacteria in a potentially changing scenario has not been studied. For example, whether less species abundance leads to decreased immune reactivity, or if smoking reduction that leads to improved immune response reduces abundance is unknown. We also do not know if or how the levels of antibodies against oral bacteria fluctuate in the long-term.

The aim of the present study was to evaluate temporal trends in the adaptive immune response to a panel of oral bacterial species. Thus, levels of systemic antibodies for 26 species of oral bacteria were examined. Plasma samples from a total of 888 unique individuals were analyzed. Biobank samples from 30-year-old women (Maternity cohort, *n* = 516) and 50-year-old women and men (Västerbotten Intervention Program (VIP) cohort, *n* = 372) in northern Sweden were randomly selected to represent cross-sectional samples for each year between 1976 and 2018.

## 2. Results

### 2.1. Study Group Characteristics

Medical and lifestyle information was collected at each screening visit in the VIP. Over the study period, the proportion of never-smokers increased continuously, whereas mean body mass index (BMI) and energy intake from fat increased, and intake of carbohydrates and vitamin C decreased (Table 1). The trend in the VIP-based study group followed that of the entire VIP cohort (Table 1). No information was available for the women in the Maternity cohort other than age and that they were less than three-months pregnant.

### 2.2. Overall Plasma Antibody Response Profiles for Oral Bacteria

**Figure 1:**
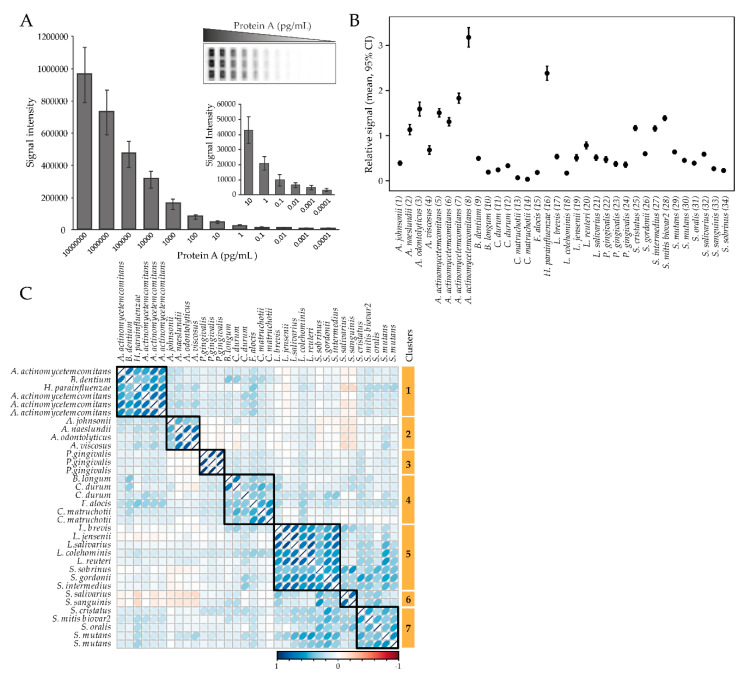
Overall antibody responses to the test panel of oral bacteria. (**A**) Checkerboard sensitivity evaluation using Protein A revealed linear detection from 10^−1^ to 10^7^ pg/mL. (**B**) Relative antibody response levels among the tested bacterial probes. Data are presented as mean and 95% CI. (**C**) Correlation-based clustering of plasma antibody levels for the tested oral bacteria panel.

The levels of plasma antibodies against a panel of 54 oral bacterial strains (Supplementary file Table S1) in 9 genera and 26 species were screened as single strains or in pools of 2–5 strains in the same species (total 34 “bacterial probes”) using a multiplex checkerboard immunoblotting assay (Figure 1A). The antibody responses varied significantly between the test probes, with the most pronounced responses for *A. actinomycetemcomitans* and *Haemophilus parainfluenzae*, and the least pronounced response for *Corynebacterium matruchotii* (Figure 1B). Clustering of bacterial antibody responses based on their correlation structure revealed seven clusters (Figure 1C). Cluster 1 (C1) consisted of antibody responses to *A. actinomycetemcomitans* (*n* = 4), *Bifidobacterium dentium*, and *H. parainfluenzae*; C2 against *Actinomyces spp*. (*A. jonsoni*, *A. naeslundii*, *A. odontolyticus*, and *A. viscosus*); C3 against *P. gingivalis* (*n* = 3); C4 against *Bifidobacterium longum*, *C. matruchotii* (*n* = 2), *Corynebacterium durum* (*n* = 2), and *F. alocis*; C5 against *Lactobacillus* spp. (*L. brevis*, *L. jensenii*, *L. salivarius*, *L. colehominis, L. reuteri*), *Streptococcus gordonii*, *Streptococcus intermedius*, and *Streptococcus sobrinus*; C6 against *Streptococcus salivarius* and *Streptococcus sanguinis*; and C7 against *Streptococcus spp*. (*S. cristatus*, *S. mitis* biovar2, *S. oralis*, and *S. mutans* (*n* = 2)) (Figure 1C).

### 2.3. Age-Related Shifts in Overall Plasma Antibody Profiles

First, the overall pattern of the antibody responses to the bacterial panel was compared between the sexes using non-parametric multivariate analysis. This was done among the 50-year-olds with men and women equally represented (Table 1) and showed no significant difference between the sexes (*p* = 0.361). Therefore, further analyses involving the 50-year-olds were performed with the sexes combined unless stated otherwise.

**Figure 2:**
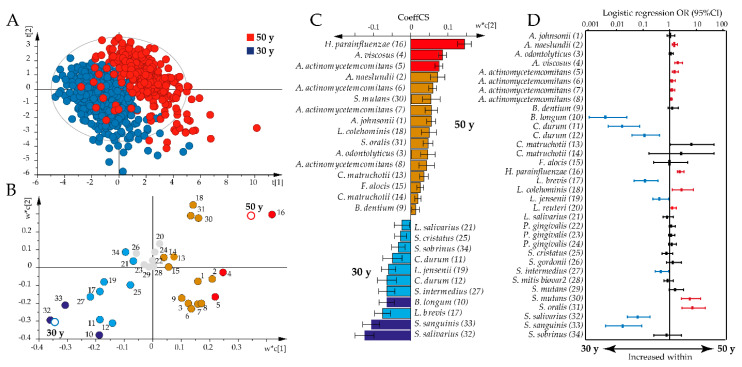
Age-dependent antibody responses to the oral bacteria test panel. Partial least square analyses with antibody responses as independent x-variables modeled against age as y were used to explore age associations with antibody profiles. (**A**) Partial least square (PLS) scatter plot (each symbol represents a participant) shows clustering of 30-year-olds versus 50-year-olds based on the concerted antibody profile. (**B**) PLS loading plot illustrating the association between each bacteria sample (colored dots, for numbers see Supplementary file Table S1) and age (open rings). Gray dots indicate non-significant x-variables. (**C**) Bar graph showing significant PLS correlation coefficients (CoeffCS) in the 50-year-old and 30-year-old groups. Red and dark blue colors indicate associations that are significant according to both the variable in projection score (B) and the PLS correlation coefficient (C), whereas orange and light blue colors indicate associations that are significant according to the PLS correlation coefficient only. (**D**) Odds ratios (ORs) with 95% confidence intervals (CIs) in the 50-year-old or 30-year-old age groups based on binary logistic regression with an antibody response to each of the 34 bacterial samples, including sex, smoking, and storage time as covariates. The red color indicates antibody responses that were significantly increased among the 50-year-olds and the blue color those that were increased among the 30-year-olds. Significance was considered for *p*-values < 0.05 with false discovery rate (FDR) after adjusting for multi-comparison using the Benjamini Hochberg correction.

However, non-parametric multivariate analysis revealed that the antibody pattern differed significantly by age between the 30-year-old and all 50-year-old participants (*p* = 0.0001) and when restricted to 30-year-old and 50-year-old women (*p* = 0.0001). Partial least square (PLS) analysis supported a shift in the overall antibody pattern between the 30-year-olds and all 50-year-olds (R^2^ = 73%, Q^2^ = 71%; Figure 2A). Thus, the 50-year-old participants were associated with increased immune responses against 17 bacteria, with the highest variable in projection scores and loading plot correlation coefficients for *H. parainfluenzae*, *A. viscosus,* and *A. actinomycetemcomitans* (Figure 2B,C). The 30-year-olds were associated with increased antibody responses against 11 bacteria, with the highest variable in projection scores and loading plot correlation coefficients for *S. salivarius* and *S. sanguinis* (Figure 2B,C).

Sensitivity analysis using binary logistic regression, adjusting for sex, smoking, and storage time, confirmed most of the associations identified by PLS; higher antibody responses were observed against *A. naeslundii, A. viscosus*, *A. actinomycetemcomitans*, *H. parainfluenzae*, *L. colehominis*, *L. reuteri*, *S. mutans*, and *Streptococcus oralis* in the 50-year-olds, and against *B. longum*, *C. durum*, *L. brevis*, *L. jensenii*, *S. intermedius*, *S. salivarius*, and *S. sanguinis* in the 30-year-olds (Figure 2D).

### 2.4. Forty-Three-Year Temporal Shifts in the Global Plasma Antibody Response to Oral Bacteria

**Figure 3:**
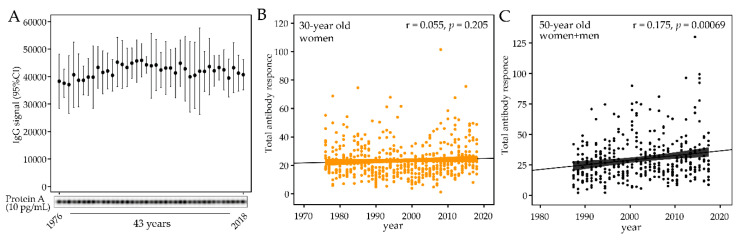
Sample storage stability and temporal trends in total antibody levels for the test panel of oral bacteria. (**A**) IgG response to Protein A (10 pg/mL) in 39 samples representing 39 of the 43 study years. Each sample consisted of 12 pooled plasma samples from the year the pooled sample represented. (**B**) Total antibody content of the panel of test bacteria in plasma samples collected from 1976 to 2018 from 30-year old men, and (**C**) from 1987 to 2017 from 50-year-old men and women. Each dot represents an individual subject and the lines indicate the trend from linear regression and 95% CI.

To evaluate whether storage time per se affected the antibody activity, 12 samples from the same year were pooled to cover the entire study period of 1976 through 2018 and analyzed against quadruplicates of serial dilutions of Protein A. To allow the samples to be run at the same time, to avoid any possible batch difference, the number of samples was limited to represent 39 of the 43 sample years. This was done by excluding samples from the years 1982, 1992, 2002, and 2012. No significant difference or trend was seen among the sample years (Figure 3A).

After storage stability was confirmed, the temporal trend of the total antibody response to the bacteria panel was evaluated. No trend was found among the 30-year-old women (1976 to 2018, *p* = 0.159, Figure 3B), but the total titer increased over time in the 50-year-old participants (1987 to 2017 *p*_all_ = 0.0003, Figure 3C). The increasing trend was consistent among 50-year old men and women (*p*_men_ = 0.0008 and *p*_women_ = 0.005).

Next, the global pattern of antibody responses to the 34 bacterial probes were compared over time using non-parametric multivariate analysis. This indicated a time-dependent profile shift in both age groups (*p*_30-year_ = 0.0001 and *p*_50-year_ = 0.0003). The global antibody pattern did not differ by BMI (*p* = 0.637), education level (*p* = 0.273), or smoking status (*p* = 0.965) when analyzed among the present 50-year-old participants.

**Figure 4:**
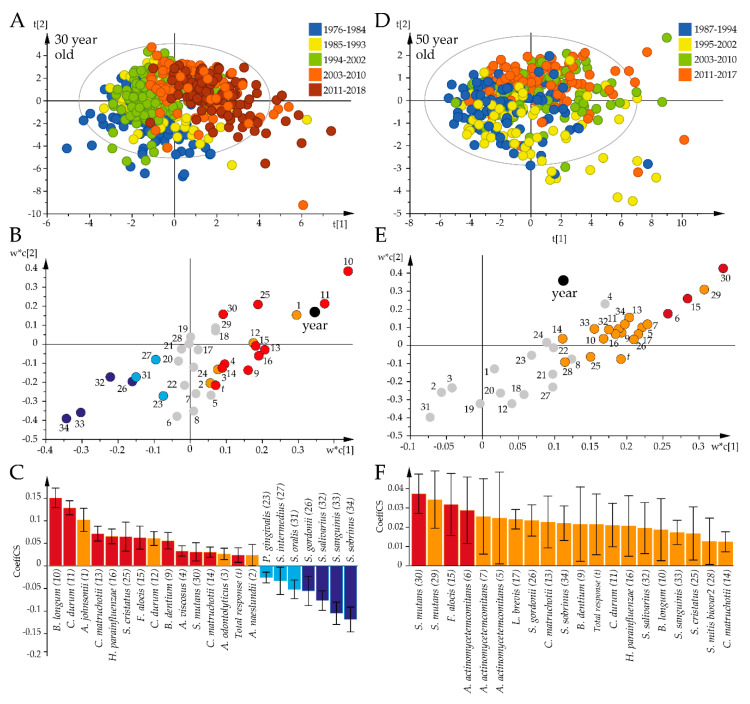
Temporal trends in the global bacterial antibody response profiles for the 30-year-old and 50-year-old participants. Results from PLS modeling with antibody responses as independent x-variables and time periods as dependent variables are illustrated in a loading scatter plot (indicating subject distribution), loading plot (indicating sample distribution), and PLS correlation coefficient plot (indicating mean correlations with 95% CI). (**A**–**C**) The 30-year-olds. (**D**–**F**) The 50-year-olds. Red/orange bars indicate significantly increased and blue/light blue significantly decreased responses over time. Red and dark blue coloring indicate significant associations according to both the variable projection scores and the PLS correlation coefficients, whereas orange and light blue coloring indicate significance according to the PLS correlation coefficient only. Gray dots in (**B**) and (**E**) indicate non-significant x-variables.

The identified time-related shift in the global antibody pattern was followed up by PLS analysis to identify the species that were influential over the study period among the 30-year-old (Figure 4A) and 50-year-old participants (Figure 4B). Both PLS models were strong (R^2^ = 67%, Q^2^ = 58%; Figure 4A vs. R^2^ = 43%, Q^2^ = 25%; Figure 4B). PLS regression indicated increasing antibody responses to 15 bacterial species associated with progressing time among the 30-year-olds, with the most prominent being *B. longum*, *C. durum*, *A. johnsonii*, *C. matruchotii*, and *H. parainfluenzae*, and decreasing titers for *S. sobrinus*, *S. sanguinis*, *S. salivarius*, and *S. gordonii* (Figure 4C). Among the 50-year-old participants, progressing time was associated with increasing antibody responses to *S. mutans*, *F. alocis,* and *A. actinomycetemcomitans* (Figure 4F).

### 2.5. Species-Specific Antibody Shifts over Time

**Figure 5:**
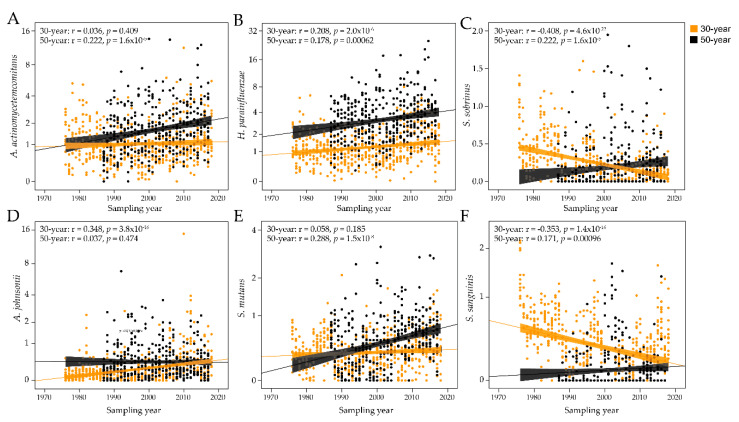
Temporal trends in antibody responses to select bacterial species. Scatter plots of the antibody responses over time for the 30-year-old and 50-year-old groups for (**A**) *Aggregatibacter* *actinomycetemcomitans*, (**B**) *Haemophilus parainfluenzae*, (**C**) *Streptococcus sobrinus*, (**D**) *Actinomyces johnsonii*, (**E**) *Streptococcus mutans*, and (**F**) *Streptococcus* *sanguinis*. The dots represent individual subjects and the lines trends with 95% CI.

The PLS results were pursued in sensitivity analyses of each of the 34 bacterial probes with generalized linier modeling, allowing adjustment for sex and smoking, as well as age when all samples were evaluated together. When employing all 888 sampling occasions, 6 antibody titers decreased over the 43-year study period (*S. gordonii*, *S. intermedius*, *S. oralis*, *S. salivarius*, *S. sanguinis*, and *S. sobrinus*) and 11 increased (*A. johnsonii*, *A. viscosus*, *A. actinomycetemcomitans*, *B. dentium*, *B. longum*, *C. durum*, *C. matruchotii*, *F. alocis*, *H. parainfluenzae*, *S. cristatus*, and *S. mutans;* Table 2). When stratifying the analyses by age, both age groups had significantly (or borderline significant) increased antibody responses over time for *A. viscosus, B. dentium, B. longum*, *C. durum, C. matruchotii, F. alocis, H. parainfluenzae, S. cristatus,* and *S. mutans*, and decreasing responses to *S. oralis.* Inverse but significant, antibody trends were observed for *S. gordonii, S. salivarius,* *S. sanguinis,* and *S. sobrinus.* Age-specific increasing time trends were observed for *A. johnsonii* and *A. odontolyticus* (30-year-olds) and *A. actinomycetemcomitans* and *L. brevis* (50-year-olds), and a decreasing trend for *S. intermedius* (30-year-olds). A panel of antibody responses by time is shown in Figure 5A–F.

## 3. Discussion

In the present study, we examined the prevalence and temporal trends of systemic immune reactivity against a collection of bacterial strains comprising 26 species in 9 genera, and representing commensal and opportunistic bacterial species in the oral cavity. We demonstrated that temporal drifts occur in global antibody profiles at the population level without any specifically noted exposure or socio-economic event, contributed novel information on age-independent and age-specific temporal trends in the antibody responses to oral bacteria, and confirmed the previously reported age-dependence in cross-sectionally determined antibody levels and profiles. Among the most striking temporal transitions in relation to oral diseases were increasing antibody levels against *A. actinomycetemcomitans*, *F. alocis,* and *S. mutans*.

The strongest antibody responses were found against *A. actinomycetemcomitans* and *H. parainfluenza* organized in the *Pasteurellaceae* family. *Pasteurellaceae* encompasses both commensal and opportunistic species of considerable ecological and medical importance [21]. Both bacterial species are found in tooth biofilms, but are prevalent in biofilms on the oral mucosa. *A. actinomycetemcomitans* is closely linked to aggressive forms of periodontitis in young individuals [22], whereas *H. parainfluenzae* is reported to be more prevalent in periodontally healthy subjects [23]. Both species have been associated with systemic diseases, such as endocarditis [24]. Generally, immunoreactivity to *A. actinomycetemcomitans* and *H. parainfluenza* was higher among the 50-year-olds than the 30-year-olds, but levels of *A. actinomycetemcomitans* antibody increased in the middle-aged group only, whereas levels of *H. parainfluenza* antibodies increased over time in both age groups.

*F. alocis* is a bacterial species that, alone or together with *A. actinomycetemcomitans,* has been identified as an indicator of periodontitis risk [10,25]. Immunoreactivity to *F. alocis* was also highest among the 50-year-olds, but immunoreactivity increased over time in both age groups. In contrast, immunoreactivity to *P. gingivalis,* yet another major pathogen in adult periodontitis [6], did not differ by age or time. Concerning caries-associated bacteria, immunoreactivity to *S. mutans, B. dentium,* and *B. longum* [18,26] increased significantly over time in both age groups, but with varying relation to age. Regrettably, we did not include *Scardovia wiggsiae,* an emerging caries “pathogen” in the test panel. Commensal streptococci were tested though, and most exhibited decreasing immunoreactivity over time in both age groups or the 30-year-old group only.

We anticipate that the generally higher immune responses to oral opportunistic species among the older participants compared to the younger participants reflect age-related dental disease progression and the associated microbiota profile in the underlying population [27,28]. However, it is less clear why the antibody levels of several dental disease-associated bacteria increased over time in cross-sectional samples in the same age stratum. This is especially noteworthy, as a significant reduction in caries and periodontitis has occurred and attendance of regular dental care increased in the targeted population over the study period. Systemic antibody responses to colonizing or invading bacteria indicate activation of the host response to current or past presence of a species [29,30]. One potential hypothesis behind a changed systemic antibody response would be a change in the abundance of the triggering bacterium, but it is not obvious that the levels of immunoreactivity reflect the bacterial load in the oral cavity [31]. From this perspective, it should be noted that we examined immunoreactivity to the oral microbiota and have no information on the bacterial load at the different time points. One more factor that may have contributed to an increased immune response may be the increase in the number of remaining teeth as a consequence of improved dental care and health [32,33]. Tooth biofilm colonizing bacteria were provided more surfaces to colonize and possibly increased in abundance [34]. In addition, smoking, which is reported to suppress the immune response [16], decreased significantly in the population over the study period [20]. We hypothesize that the recovered immune response from smoking cessation combined with altered microbiota profiles from contemporary changes in the oral condition may, at least in part, have contributed to the seemingly contradictory association between oral health and the immune response, and that this could not be seen among the 30-yeral-olds due to less variation in tooth numbers and the bacterial profile.

Lifestyle changes are reported to affect the diversity of the gastrointestinal microbiota with effects on immune responses and general health [27]. In addition, age, sex, oral conditions, and some systemic diseases have been reported to be associated with the composition and alterations of the saliva microbiota [35]. In line with this, we found distinct clustering of the 30-year-old versus 50-year-old participants based on their overall microbiota profile, as well as in the respective temporal trends, though there was no difference in the total amounts of IgG reacting with oral bacteria. We could not demonstrate any overall association with BMI, smoking, or sex in the present study. However, these associations could only be performed among the 50-year-old men and women when phenotypical data were available, leading to under-powered per year analyses and sensitivity analyses for possible interactions, e.g., smoking that subsided during the study period.

The strengths of the present study include its long follow-up period, that the plasma samples were collected under highly standardized conditions with short transfer times and long-term storage at −80 °C, and that a comparably wide panel of oral species and strains was included in the test panel. Another strength was that both the 30-year-old and 50-year-old participants were nested in population-based cohorts, i.e., the 30-year-old women were recruited at a mandatory screening for rubella in early pregnancy, and the 50-year-old men and women were recruited at a health screening (VIP) targeting all middle-aged inhabitants of the catchment area. In the VIP, the average participation rate has varied slightly over time, but with an average of 60% [20], and social selection bias, i.e., with respect to income, age, or unemployment, is reported to be insignificant [36].

This study also has some limitations that should be considered when the results are evaluated. First, though the participants were recruited from population-based screenings, which allows extrapolation to the population level in socio-economically similar communities, the results are most likely not valid beyond that. Some may also argue that the study not being a true longitudinal study, in the sense that the participants represented annual cross-sectional samples, is a limitation. However, it may be challenging to distinguish temporal trends from aging effects per se in a strict longitudinal study. Furthermore, we are not aware of any biobank that has collected and stored high quality samples collected annually from the same individuals, and such a group would likely be severely biased, as only the fittest would survive from 30 years to 90+ years of age. From a methodological standpoint, using whole bacterial cells for the antibody screening means a higher risk of cross-reactivity among species/strains carrying the same cell surface immune-reactive epitopes. In addition, pooling a few strains in some of the bacterial probes was a balance to limit the number of runs and potential batch variation. We have not performed any targeted analyses, but they should be included in follow-up studies as suggested below.

The immune response of individuals to bacteria, including those in the mouth, is generally anticipated to be a reflection of age, bacterial load, host traits (including genetics), lifestyle, and general and local health. Thus, shifts in the immune profile should mirror a shift in one or more of these factors, at least at the group level. As the present study was restricted to a description of the antibody profiles over time, follow-up studies may focus on targeted analyses of the most striking species that were found to change over the study period using species-targeted ELISAs or other suitable methods, combined with oral microbiota characterization using multiplex sequencing, clinically assessed oral health, and general host traits. These analyses will contribute to deepen knowledge and possibly target potential causal associations.

Based on the findings of the current study, we conclude that shifts have occurred in the global antibody profiles and shifts to defined species over the four decades we evaluated followed. During this period, there were significant improvements in oral hygiene procedures, caries and periodontal status, and number of teeth in the mouth, as well as smoking prevalence, which has decreased significantly. Notably, we found a temporal increase in the immune response to *A. actinomycetemcomitans*, *F. alocis,* and *S. mutans,* which may seem unexpected given the overall reduction in dental disease, but the total bacterial load may have increased with the number of teeth, and immunoreactivity increased as smoking ceased. In addition, depletion of the commensal microflora may contribute to promoting the growth of certain species. However, this study does not allow any conclusions to be made on potential associations between the antibody responses and potential oral or general host trait determinants, and these aspects should be explored in follow-up studies.

## 4. Materials and Methods

### 4.1. Study Group

IgG levels for oral bacterial species were analyzed in plasma samples originating from two independent cohorts in northern Sweden. Plasma samples were retrieved from the Northern Sweden Biobank, where they had been deposited within a few hours after collection and stored at −80 °C [37].

In the Maternity cohort, women who were in the first trimester of pregnancy donated blood to the biobank in conjunction with the mandatory test for rubella antibodies. Twelve samples from 30-year-old women were randomly selected each year between 1976 and 2018 (516 samples total, representing 43 consecutive years). In the VIP, 40-, 50-, or 60-year-old men and women were invited to a health screening including collection of lifestyle and medical information and blood. Twelve plasma samples were randomly selected each year between 1987 and 2017 (372 samples total, representing 31 consecutive years). This generated a set of 888 plasma samples collected between 1976 and 2018.

The study was approved by the Regional Ethical Board at Umeå University, Sweden (Dnr 2018-231-31 M).

### 4.2. Bacteria and Culturing

Bacterial strains were grown for 48 hours on Columbia-based blood agar, Rogosa, or chocolate agar plates under aerobic or anaerobic conditions at 37 °C as indicated in Table S1. Bacteria were harvested using sterilized cotton swabs, re-suspended, washed twice in 50 mM Tris-HCl (pH 7.5) with 150 mM NaCl (1× TBS), and adjusted to an optical density of 1.0 at 600 nm before storing at −80 °C in 500 µL aliquots.

### 4.3. Antibody Detection

Antibodies were detected by a multiplex immunoblotting assay in a checkerboard device as described previously [38]. Briefly, a nitrocellulose membrane (Amersham™ Protran® GE10600003, Merck, Solna, Sweden) pre-wetted (18 Ω Milli-Q) and equilibrated in 1× TBS for 10 min was loaded in a Miniblotter device (Miniblotter 45MN, Interchime, Montlucon Cedex, France) according to the manufacturer´s instructions. Bacterial suspensions (150 µL), positive controls (Protein A at 0.005–1 µg/mL; P6031, Merck, Solna, Sweden), and negative controls (TBS) were loaded into the lanes of the Miniblotter and the gasket sealed with adhesive film to avoid evaporation. The device was rotated slowly for 1 h at room temperature followed by overnight incubation at 4 °C on a balanced table. The liquids were removed from the lanes by vacuum suction and washed with 500 µL TBS with 0.1% Tween 20 (TBS-T). Afterwards, the membrane was removed from the Miniblotter, rinsed 3× for 1 min in TBS-T prior to blocking in TBS-T containing 5% blocking reagent (ECL™ Advance Blocking Reagent, GERPN418, Merck, Solna, Sweden) for 1 h at room temperature with rotation. The Miniblotter was re-assembled, plasma applied perpendicular to the bacteria, sealed with adhesive film, and slowly rotated for 1 h at room temperature. The liquids were removed by vacuum suction and washed once with 500 µL TBS-T. The membrane was removed and rinsed 3× for 1 min in TBS-T before treatment with 0.3% H_2_O_2_ for 10 min (H1009, Merck, Solna, Sweden) to reduce endogenous bacterial peroxidase activity. This was followed by rinsing and washing the membrane once for 15 min and 3× for 5 min with TBS-T before incubation with an anti-human-IgG-Fab peroxidase-labeled secondary antibody (Anti-Human-IgG-Fab, A0293, Merck, Solna, Sweden) diluted in TBS-T with 5% ECL™ Advance Blocking Reagent (GE Healthcare, RPN418, Merck, Solna, Sweden) for 1 h at room temperature with slow rotation. Finally, the membrane was rinsed twice, washed once for 15 min and 3× for 5 min with TBS-T before signal development (ECL™ Prime Western Blotting Detection Reagent, GERPN2236, Merck, Solna, Sweden). Signals were detected using the ChemiDoc^TM^ XRS+ System (BioRad, Solna, Sweden). The 888 plasma samples were run in 28 batches, with median signals normalized to adjust for batch to batch variation. Bacteria suspensions were either for a single bacterial strain or pools of 2–5 strains in the same species (Supplementary file Table S1). Pooling of bacterial strains was based on pilot testings where the pooled strains gave the same immune response for the individual.

### 4.4. Estimation of IgG Storage Stability

To evaluate the storage stability of IgG over the 43-year study period, the 12 samples from the Maternity cohort were pooled for a comparative analysis. These samples were selected because they covered the entire study period compared to VIP, which only covered 31 years. The decision to exclude 4 years (1982, 1992, 2002, and 2012) was due to the maximum loading capacity of the checkerboard assay, which is 39 samples in addition to the controls. Thus, these samples could be analyzed in the same run and batch variation excluded. The pooled samples were diluted with TBS (1:500) and evaluated for binding against four separate (quadruplet) Protein A samples (10 pg/mL). Sample loading, washing, and detection were performed as described in Section 4.3.

### 4.5. Data Handling and Statistical Analyses

The SPSS software version 25.0 was used for descriptive statistics, including odds ratios (ORs) with 95% confidence intervals (CIs). Group comparisons of continuous variables were performed using the Mann–Whitney U test, and for categorical variables the chi^2^ test. Correlation structures and cluster formation were obtained in R using the corrplot package [39]. Binary regression analysis was used to evaluate differences between 30- and 50-year-old subgroups with adjustments for sex, smoking, and storage time. PLS modeling (SIMCA 15, Sartorius Stedim Data Analytics AB, Malmö, Sweden) was used to illustrate differences between subgroups based on their bacterial antibody profile. The PLS models included age group or sex as dependent variables (y-variables) against a swarm of independent x-variables (i.e., the antibody responses against the 34 bacterial probes). PLS results were displayed in loading plots. Variables utilized for PLS regression were auto-scaled and logarithmically transformed as needed to improve normality. Generalized linear models were used for sensitivity analyses to evaluate temporal antibody trends by age group. All models were adjusted for sex and smoking, as well as age when the whole samples (*n* = 888) were evaluated. All tests were two-tailed. Correction for multiple comparisons was done by the Benjamini Hochberg FDR. *p*-values were considered significant at FDR < 0.05.

## Figures and Tables

**Table 1:** Study group characteristics for the 50-year-old men and women in the Västerbotten Intervention Program (VIP)-based study group and the basic VIP cohort. Data are presented as mean (95% confidence interval) or percent at screening.

	VIP Cohort		Present Study Group		
	*n* = 110,663		*n* = 372	Temporal Trend	
	Measure	Trend	Measure	Trend	*p*-Value
Sample years	1990–2017	-	1987–2017	-	-
Proportion women	50.7%	-	50.0%	-	-
University level education	30.4%	↑	30.6%	↑	0.03
BMI ^a^, kg/m^2^	26.6 (26.5, 26.6)	↑	26.1 (25.7, 26.4)	↑	0.018
Fasting blood glucose ^a^, mol/L	5.52 (5.51, 5.53)	→	5.29 (5.21, 5.37)	→	0.149
Never-smoker	39.2% to 50.7%	↑	41.7% to 71.4%	↑	0.004
Nutrient intake ^a^					
Total fat, E%	35.8 (35.7, 35.9)	↑	35.4 (34.7, 36.1)	↑	<0.001
Saturated fat, E%	14.9 (14.9, 15.0)	↑	14.8 (14.3, 15.2)	↑	<0.001
Carbohydrates, E%	46.5 (46.4, 46.5)	↓	46.6 (45.9, 47.4)	↓	<0.001
Sucrose, E%	5.84 (5.82, 5.85)	↓	5.77 (5.50, 6.05)	↓	<0.001
Protein, E%	15.1 (15.1, 15.1)	→	14.8 (14.6, 15.0)	→	0.044
Vitamin C, mg/day	69.9 (69.6, 70.1)	↓	72.8 (68.2, 77.5)	↓	0.017
Vitamin D, mg/day	5.22 (5.21, 5.23)	→	5.36 (5.15, 5.57)	→	0.152
Vitamin E, mg/day	6.07 (6.06, 6.08)	↓	6.08 (5.92, 6.24)	→	0.799

^a^ Adjusted for sex and education level by general linear modeling. Mean nutrient intakes were also adjusted for total energy intake.

**Table 2:** Trends of bacterial antibody response profiles over time. Generalized linear models were used to evaluate antibody response over time. All models were adjusted for sex and smoking, as well as age when the whole sample (*n* = 888) was evaluated. Three groups were evaluated, the 30-year-olds and 50-year-olds together (total sample), and each independently. *p*-values below an FDR of 0.05 using Benjamini Hochberg correction are shown in bold.

	Sample Collections					
	All Samples (*n* = 888)		30-Year-Olds (*n* = 516)		50-Year-Olds (*n* = 372)	
Species (No.)	β-Value	*p*-Value	β-Value	*p*-Value	β-Value	*p*-Value
*A. johnsonii (1)*	0.009	<0.0005	0.013	<0.0005	−0.002	0.518
*A. naeslundii (2)*	0.003	0.477	0.006	0.097	−0.004	0.792
*A. odontolyticus (3)*	0.011	0.117	0.015	0.009	−0.002	0.933
*A. viscosus (4)*	0.014	<0.0005	0.007	0.001	0.033	0.013
*A. actinomycetemcomitans (5)*	0.014	<0.0005	0.006	0.159	0.036	<0.0005
*A. actinomycetemcomitans (6)*	0.005	0.272	−0.007	0.231	0.038	<0.0005
*A. actinomycetemcomitans (7)*	0.015	0.009	0.004	0.549	0.046	<0.0005
*A. actinomycetemcomitans ^a^ (8)*	0.012	0.205	0.000	0.990	0.045	0.024
*B. dentium (9)*	0.007	<0.0005	0.006	0.001	0.009	0.029
*B. longum (10)*	0.011	<0.0005	0.014	<0.0005	0.002	0.010
*C. durum (11)*	0.011	<0.0005	0.014	<0.0005	0.003	0.001
*C. durum (12)*	0.004	<0.0005	0.005	<0.0005	0.001	0.548
*C. matruchotii (13)*	0.002	<0.0005	0.001	< 0.0005	0.003	<0.0005
*C. matruchotii (14)*	0.001	0.006	0.000	0.107	0.002	0.021
*F. alocis ^a^ (15)*	0.003	<0.0005	0.002	< 0.0005	0.006	<0.0005
*H. parainfluenzae (16)*	0.029	<0.0005	0.016	<0.0005	0.066	<0.0005
*L. brevis (17)*	0.001	0.586	0.000	0.982	0.005	0.010
*L. colehominis (18)*	0.001	0.245	0.001	0.283	0.001	0.582
*L. jensenii (19)*	−0.001	0.557	−0.002	0.584	−0.001	0.829
*L. reuteri (20)*	−0.002	0.404	−0.004	0.089	0.003	0.744
*L. salivarius (21)*	0.000	0.927	−0.002	0.461	0.007	0.156
*P. gingivalis (22)*	0.003	0.341	0.002	0.576	0.005	0.384
*P. gingivalis (23)*	−0.001	0.815	−0.001	0.741	0.001	0.916
*P. gingivalis (24)*	0.002	0.380	0.002	0.387	0.002	0.724
*S. cristatus (25)*	0.011	<0.0005	0.011	<0.0005	0.012	0.048
*S. gordonii ^a^ (26)*	−0.003	0.012	−0.008	<0.0005	0.009	0.010
*S. intermedius (27)*	−0.005	0.026	−0.008	0.001	0.004	0.300
*S. mitis ^a^ (28)*	0.004	0.123	0.002	0.428	0.008	0.095
*S. mutans ^a^ (29)*	0.008	<0.0005	0.003	0.024	0.022	<0.0005
*S. mutans (30)*	0.005	<0.0005	0.002	0.026	0.014	<0.0005
*S. oralis ^a^ (31)*	−0.004	<0.0005	−0.004	<0.0005	−0.004	0.053
*S. salivarius (32)*	−0.012	<0.0005	−0.017	<0.0005	0.004	0.014
*S. sanguinis ^a^ (33)*	−0.007	<0.0005	−0.010	<0.0005	0.002	0.041
*S. sobrinus (34)*	−0.006	<0.0005	−0.009	<0.0005	0.005	0.003

^a^ Indicates a pool of strains as described in Table S1.

## Supplementary Materials

The following supplementary material is available online at https://www.mdpi.com/2076-0817/9/7/544/s1, Table S1: Bacterial strains used for immunoblotting in the checker board assay.

## References

[1] Dewhirst F.E., Chen T., Izard J., Paster B.J., Tanner A.C., Yu W.H., Lakshmanan A., Wade W.G. (2010). The human oral microbiome. J. Bacteriol..

[2] Kim D., Barraza J.P., Arthur R.A., Hara A., Lewis K., Liu Y., Scisci E.L., Hajishengallis E., Whiteley M., Koo H. (2020). Spatial mapping of polymicrobial communities reveals a precise biogeography associated with human dental caries. Proc. Natl. Acad. Sci. USA.

[3] Sanz M., Beighton D., Curtis M.A., Cury J.A., Dige I., Dommisch H., Ellwood R., Giacaman R.A., Herrera D., Herzberg M.C. (2017). Role of microbial biofilms in the maintenance of oral health and in the development of dental caries and periodontal diseases. Consensus report of group 1 of the Joint EFP/ORCA workshop on the boundaries between caries and periodontal disease. J. Clin. Periodontol..

[4] Curtis M.A., Diaz P.I., Van Dyke T.E. (2020). The role of the microbiota in periodontal disease. Periodontology 2000.

[5] Deo P.N., Deshmukh R. (2019). Oral microbiome: Unveiling the fundamentals. J. Oral Maxillofac. Pathol..

[6] Dahlen G., Basic A., Bylund J. (2019). Importance of Virulence Factors for the Persistence of Oral Bacteria in the Inflamed Gingival Crevice and in the Pathogenesis of Periodontal Disease. J. Clin. Med..

[7] Esberg A., Sheng N., Marell L., Claesson R., Persson K., Boren T., Stromberg N. (2017). *Streptococcus Mutans* Adhesin Biotypes that Match and Predict Individual Caries Development. EBioMedicine.

[8] Belibasakis G.N., Maula T., Bao K., Lindholm M., Bostanci N., Oscarsson J., Ihalin R., Johansson A. (2019). Virulence and Pathogenicity Properties of *Aggregatibacter actinomycetemcomitans*. Pathogens.

[9] Hajishengallis G. (2015). Periodontitis: From microbial immune subversion to systemic inflammation. Nat. Rev. Immunol..

[10] Fine D.H., Markowitz K., Fairlie K., Tischio-Bereski D., Ferrendiz J., Furgang D., Paster B.J., Dewhirst F.E. (2013). A consortium of *Aggregatibacter actinomycetemcomitans*, *Streptococcus parasanguinis*, and *Filifactor alocis* is present in sites prior to bone loss in a longitudinal study of localized aggressive periodontitis. J. Clin. Microbiol..

[11] Akrivopoulou C., Green I.M., Donos N., Nair S.P., Ready D. (2017). *Aggregatibacter actinomycetemcomitans* serotype prevalence and antibiotic resistance in a UK population with periodontitis. J. Glob. Antimicrob. Resist..

[12] Tjokro N.O., Kittichotirat W., Torittu A., Ihalin R., Bumgarner R.E., Chen C. (2019). Transcriptomic Analysis of *Aggregatibacter actinomycetemcomitans* Core and Accessory Genes in Different Growth Conditions. Pathogens.

[13] Ebersole J.L., Dawson D.R., Morford L.A., Peyyala R., Miller C.S., Gonzalez O.A. (2013). Periodontal disease immunology: ‘Double indemnity’ in protecting the host. Periodontol 2000.

[14] Ebersole J.L., Dawson D.A., Emecen Huja P., Pandruvada S., Basu A., Nguyen L., Zhang Y., Gonzalez O.A. (2018). Age and Periodontal Health - Immunological View. Curr. Oral Health Rep..

[15] Johansson A., Eriksson M., Ahren A.M., Boman K., Jansson J.H., Hallmans G., Johansson I. (2011). Prevalence of systemic immunoreactivity to *Aggregatibacter actinomycetemcomitans* leukotoxin in relation to the incidence of myocardial infarction. BMC Infect. Dis..

[16] Michaud D.S., Izard J., Rubin Z., Johansson I., Weiderpass E., Tjonneland A., Olsen A., Overvad K., Boutron-Ruault M.C., Clavel-Chapelon F. (2013). Lifestyle, dietary factors, and antibody levels to oral bacteria in cancer-free participants of a European cohort study. Cancer Causes Control.

[17] Lif Holgerson P., Esberg A., Sjödin A., West C.E., Johansson I. (2020). Salivary microbiota transformation from 2 days to 18 years of age using 16S ribosomal RNA gene sequencing. Sci. Rep..

[18] Eriksson L., Lif Holgerson P., Johansson I. (2017). Saliva and tooth biofilm bacterial microbiota in adolescents in a low caries community. Sci. Rep..

[19] Johansson I., Witkowska E., Kaveh B., Lif Holgerson P., Tanner A.C. (2016). The Microbiome in Populations with a Low and High Prevalence of Caries. J. Dent. Res..

[20] Norberg M., Wall S., Boman K., Weinehall L. (2010). The Vasterbotten Intervention Programme: Background, design and implications. Glob. Health Act..

[21] Murra M., Lutzen L., Barut A., Zbinden R., Lund M., Villesen P., Norskov-Lauritsen N. (2018). Whole-Genome Sequencing of Aggregatibacter Species Isolated from Human Clinical Specimens and Description of Aggregatibacter kilianii sp. nov. J. Clin. Microbiol..

[22] Aberg C.H., Kelk P., Johansson A. (2015). *Aggregatibacter actinomycetemcomitans*: Virulence of its leukotoxin and association with aggressive periodontitis. Virulence.

[23] Colombo A.P., Boches S.K., Cotton S.L., Goodson J.M., Kent R., Haffajee A.D., Socransky S.S., Hasturk H., Van Dyke T.E., Dewhirst F. (2009). Comparisons of subgingival microbial profiles of refractory periodontitis, severe periodontitis, and periodontal health using the human oral microbe identification microarray. J. Periodontol..

[24] Norskov-Lauritsen N. (2014). Classification, identification, and clinical significance of Haemophilus and Aggregatibacter species with host specificity for humans. Clin. Microbiol. Rev..

[25] Hiranmayi K.V., Sirisha K., Ramoji Rao M.V., Sudhakar P. (2017). Novel Pathogens in Periodontal Microbiology. J. Pharm. Bioallied Sci..

[26] Scharnow A.M., Solinski A.E., Wuest W.M. (2019). Targeting *S. mutans* biofilms: A perspective on preventing dental caries. MedChemComm.

[27] Moossavi S., Bishehsari F. (2019). Microbes: Possible link between modern lifestyle transition and the rise of metabolic syndrome. Obes. Rev..

[28] Willis J.R., Gabaldon T. (2020). The Human Oral Microbiome in Health and Disease: From Sequences to Ecosystems. Microorganisms.

[29] Kononen E., Gursoy M., Gursoy U.K. (2019). Periodontitis: A Multifaceted Disease of Tooth-Supporting Tissues. J. Clin. Med..

[30] Oscarsson J., Claesson R., Lindholm M., Hoglund Aberg C., Johansson A. (2019). Tools of *Aggregatibacter actinomycetemcomitans* to Evade the Host Response. J. Clin. Med..

[31] Kogut M.H., Lee A., Santin E. (2020). Microbiome and pathogen interaction with the immune system. Poult. Sci..

[32] Koch G., Helkimo A.N., Ullbro C. (2017). Caries prevalence and distribution in individuals aged 3-20 years in Jonkoping, Sweden: Trends over 40 years. Eur. Arch Paediatr. Dent..

[33] Norderyd O., Koch G., Papias A., Kohler A.A., Helkimo A.N., Brahm C.O., Lindmark U., Lindfors N., Mattsson A., Rolander B. (2015). Oral health of individuals aged 3–80 years in Jonkoping, Sweden during 40 years (1973–2013). II. Review of clinical and radiographic findings. Swed. Dent. J..

[34] Kilian M., Chapple I.L., Hannig M., Marsh P.D., Meuric V., Pedersen A.M., Tonetti M.S., Wade W.G., Zaura E. (2016). The oral microbiome—An update for oral healthcare professionals. Br. Dent. J..

[35] Lira-Junior R., Akerman S., Klinge B., Bostrom E.A., Gustafsson A. (2018). Salivary microbial profiles in relation to age, periodontal, and systemic diseases. PLoS ONE.

[36] Weinehall L., Hallgren C.G., Westman G., Janlert U., Wall S. (1998). Reduction of selection bias in primary prevention of cardiovascular disease through involvement of primary health care. Scand. J. Prim. Health Care.

[37] Hallmans G., Agren A., Johansson G., Johansson A., Stegmayr B., Jansson J.H., Lindahl B., Rolandsson O., Soderberg S., Nilsson M. (2003). Cardiovascular disease and diabetes in the Northern Sweden Health and Disease Study Cohort—Evaluation of risk factors and their interactions. Scand. J. Public Health Suppl..

[38] Sakellari D., Socransky S.S., Dibart S., Eftimiadi C., Taubman M.A. (1997). Estimation of serum antibody to subgingival species using checkerboard immunoblotting. Oral Microbiol. Immunol..

[39] Wei T., Simko V. (2017). R Package “Corrplot”: Visualization of a Correlation Matrix (Version 0.84). https://github.com/taiyun/corrplot.

