# Supplementary material for: 43-Year Temporal Trends in Immune Response to Oral Bacteria in a Swedish Population
Source: Pathogens. 2020 Jul 7;9(7):544. doi: 10.3390/pathogens9070544 (PMC7400255; doi:10.3390/pathogens9070544)
Supplement: Supplementary file 1 [file pathogens-09-00544-s001.pdf]

Supplementary Table 1. Bacterial strains used in checker board assay. Bacterial strains were cultivated for 48-72 hours on blood, chocolate blood, or Rogosa agar plates under aerobic (5% CO_2) or anaerobic conditions at 37°C as indicated below. Bacterial cells were harvested using cotton swabs, re-suspended, washed twice in 50 mM Tris-HCl (pH 7.5) containing 150 mM NaCl (TBS), and adjusted to an optical density of 1.0 at 600 nm before storing at -80°C in aliquots.

Number	Species	Isolate/name	Agar plate	Condition
1	*Actinomyces johnsonii*	PK1259	chocolate	aerobic
2	*Actinomyces naeslundii*	PK606	chocolate	aerobic
3	*Actinomyces odontolyticus*	PK984	chocolate	aerobic
4	*Actinomyces viscosus*	Ly7	chocolate	aerobic
5	*Aggregatibacter actinomycetemcomitans*	D7ss (serotype a)	blood	aerobic
6	*Aggregatibacter actinomycetemcomitans*	Y4 (serotype b)	blood	aerobic
7	*Aggregatibacter actinomycetemcomitans*	NCTC 9710 (serotype c)	blood	aerobic
8	*Aggregatibacter actinomycetemcomitans*	POOL 1	blood	aerobic
9	*Bifidobacterium denticum*	CCUG 18367 T	chocolate	anaerobic
10	*Bifidobacterium longum*	CCUG 15137	chocolate	anaerobic
11	*Corynebacterium durum*	CCUG 60194	chocolate	aerobic
12	*Corynebacterium durum*	CCUG 43120	chocolate	aerobic
13	*Corynebacterium matruchotii*	CCUG 46620 T	blood	anaerobic
14	*Corynebacterium matruchotii*	CCUG 47160	blood	anaerobic
15	*Filifactor alocis*	POOL 2	blood	anaerobic
16	*Haemophilus parainfluenzae*	CCUG 12836 T	chocolate	anaerobic
17	*Lactobacillus brevis*	CCUG 30670	rogosa	anaerobic
18	*Lactobacillus colehominis*	CCUG 44007	rogosa	anaerobic
19	*Lactobacillus jensenii*	CCUG 35572	rogosa	anaerobic
20	*Lactobacillus reuteri*	CCUG 33624	rogosa	anaerobic
21	*Lactobacillus salivarius*	CCUG 31453	rogosa	anaerobic
22	*Porphyromonas gingivalis*	W381	chocolate	anaerobic
23	*Porphyromonas gingivalis*	CCUG 33277	chocolate	anaerobic
24	*Porphyromonas gingivalis*	W50	chocolate	anaerobic
25	*Streptococcus cristatus*	CCUG 43159	chocolate	aerobic
26	*Streptococcus gordonii*	POOL 3	blood	aerobic
27	*Streptococcus intermedius*	ATCC 27335	chocolate	aerobic
28	*Streptococcus mitis*	POOL 4	blood	aerobic
29	*Streptococcus mutans*	POOL 5	blood	aerobic
30	*Streptococcus mutans*	CCUG 11877 T	blood	aerobic
31	*Streptococcus oralis*	POOL 6	blood	aerobic
32	*Streptococcus salivarius*	ATCC 7073	blood	aerobic
33	*Streptococcus sanguinis*	POOL 7	blood	aerobic
34	*Streptococcus sobrinus*	OMZ176	blood	aerobic

POOL 1	389U (serotype a), IDH1705 (serotype d), 173 Gh (serotype e), C1000 (serotype f), SEA275 (serotype f)
POOL 2	CCUG 47790 and 148B-17 (clinical isolate)
POOL 3	SK120, Blackburn, M5, SK12, DL 1, SK184
POOL 4	SK34, SK113
POOL 5	UA159, NG8, LT11, Ingbritt, JBP
POOL 6	LA11, LVG1, SK143, SK2, ATCC10554
POOL 7	ATCC 10556, SK112
